# Sexual Function in Women Survivors of Hematologic Malignancy after Autologous Hematopoietic Stem Cell Transplantation

**DOI:** 10.3390/curroncol30030223

**Published:** 2023-03-01

**Authors:** Ioanna Tsatsou, Kyriaki Mystakidou, Theodoula Adamakidou, Theocharis Konstantinidis, Ioannis Kalemikerakis, Antonis Galanos, Ourania Govina

**Affiliations:** 1 Department of Nursing, University of West Attica, 12243 Egaleo, Greece; 2 Pain Relief and Palliative Care Unit, Department of Radiology, Areteion Hospital, School of Medicine, National and Kapodistrian University of Athens, 11526 Athens, Greece; 3 Department of Nursing, Hellenic Mediterranean University, 71410 Heraklion, Greece; 4 Laboratory for Research of the Musculoskeletal System, School of Medicine, National and Kapodistrian University of Athens, 14561 Athens, Greece

**Keywords:** autologous hematopoietic stem cell transplantation, hematologic malignancy, women, survivors, sexual function, female sexual function index

## Abstract

A multicenter, cross-sectional study was conducted to assess the sexual function of women survivors of hematologic malignancy after autologous hematopoietic stem cell transplantation (AHSCT), and to compare it with that of healthy women controls. Fifty-six sexually active women survivors of hematologic malignancy who underwent AHSCT were recruited through convenience sampling, as well as 60 healthy women. Demographic and clinical data questionnaires and the Female Sexual Function Index (FSFI) were completed. Survivors had a median age of 44 years and a median time since transplant of 3 years, while 48.2% had Hodgkin Lymphoma. Survivors reported an average level of sexual dysfunction, with a total score mean ± SD = 22.51 ± 8.95. The best sexual functioning domain was “pain” and the most affected was “orgasm”. There was a statistically significant association between survivors’ sexual function and age (*p* < 0.0005) in both the unifactorial and the multifactorial analysis. In addition, there was a statistically significant association between survivors’ sexual function and functional status (*p* < 0.0005), menopausal status (*p* < 0.0005), the presence of children (*p* = 0.002), education (*p* < 0.0005), and diagnosis (*p* < 0.0005). Healthy women had statistically significantly higher scores in all FSFI subscales (*p* < 0.0005). Women survivors of hematologic malignancy, treated by AHSCT, had impaired sexual function, implying the need to implement regular sexual health assessment in survivorship care.

## 1. Introduction

Hematologic malignancies are a heterogeneous group of diagnoses with varying frequencies, prognoses, and etiologies. The World Health Organization has classified hematologic malignancies into two main categories, lymphatic and myeloid diseases [1]. Hematopoietic stem cell transplantation (HSCT) is a treatment option for hematologic diseases and malignancies. Autologous hematopoietic stem cell transplantation (AHSCT), which includes the administration of the patient’s stem cells, is mainly applied to lymphomas and multiple myeloma [2].

The latest 2019 data from the European Bone Marrow Team (EBMT) annual survey showed that the number of transplantations is constantly increasing. Ιn 2019, the number of AHSCTs had a 3.1% increase and the number of patients that received one was 25.018 [3]. Survival rates for HSCT have improved significantly and this makes it necessary to address its long-term complications in survivorship.

The term “cancer survivor” usually refers to any person diagnosed with cancer, with the survivorship journey beginning at diagnosis and including all therapeutic and non-therapeutic interventions [4]. Similarly, an HSCT survivor is a person who was treated by HSCT, with survival beginning on the day of transplantation (stem cell infusion day 0) [5].

Survivors of hematologic malignancies report great concerns regarding their altered sexuality [6]. Sexual dysfunction is a frequently described problem in studies after autologous and allogeneic HSCT. Although the effect of a life-threatening disease on sexuality may not be the first concern for patients treated by HSCT, it is an important aspect of survival and quality of life that is often overlooked [7,8]. In women surviving HSCT, long-term sexual complications include decreased libido, vaginal changes, premature menopause, sex hormone dysfunction, dyspareunia, and infertility. The high dose, intensive chemotherapy for the AHSCT and irradiation contribute to sterility, sexual dysfunctions, genital tissue atrophy or scarring, and ovarian dysfunction [7,9]. Additionally, other side effects of anticancer treatments, like alopecia, fatigue, immunosuppression, diarrhea, or sadness negatively affect survivors’ sexual function [10]. In general, women report more sexual problems than men after HSCT, with worse sexual satisfaction and sexual function [7,11].

In addition to the physical consequences of transplantation, survivors face many psychosocial challenges and emotional consequences that have a negative impact on their sexuality. Problems arise in partner relationships that lead to increased distress to the survivor and the partner [12], like lack of intimacy and communication, insecurity, or changing relationship dynamics [13]. Psychological issues that can alter sexual functioning and reduce libido, as well as the ability to enjoy sex, in women after cancer, include poor body image, low self-esteem, depression, sadness, grief, anxiety, fear, or frustration [14]. Overall, the psychological distress after HSCT induces sexual dysfunction [15].

Current literature has examined sexual function after HSCT, compared to the time before transplantation or with that of the general population. Survivors report a decrease in sexual activity, satisfaction, and loss of sexual desire, even five to 10 years after HSCT [16,17,18,19]. Nevertheless, most studies assessed sexual function after allogeneic HSCT, while there is a scarcity of studies that assess sexual function after AHSCT. Assessing and increasing understanding of HSCT survivors’ long-term sexuality problems and the factors that influence them is of utmost importance for their well-being. Sexuality is recognized as an important parameter for HSCT survivors’ quality of life, even in the first years after the transplantation [11]. Thus, the purpose of this study was to evaluate the sexual function of women survivors of hematologic malignancy, treated by AHSCT, and to compare it with that of healthy matched controls.

## 2. Materials and Methods

### 2.1. Study Design

A multicenter, cross-sectional, non-randomized study was conducted. Data collection lasted from December 2019 to March 2022.

### 2.2. Setting and Sample

Through convenience sampling, 56 female survivors were recruited from the hematology units of five public hospitals in Athens. The sample was selected from survivors attending the hematology units as outpatients for their planned follow-up, after the transplantation. In total, 64 women were approached to participate in the study, of whom 8 declined to participate. Thus, the response rate was 87.5%.

Daily collaboration between the research team and the medical and nursing staff of each unit, led to the identification of survivors who met the study’s inclusion criteria. Adult and sexually active women survivors, who were treated by AHSCT from six months to five years (before the assessment of sexual function), who had not relapsed in the previous year, with functional status 0–1 (by ECOG scale; 0: functional status with normal activity without limitation, as before the disease; 1: limitation of vigorous physical activities, but normal mobility and ability for light work; 2: capable only of self-care, but not any work; 3: capable of only limited self-care; and 4: completely incapable of self-care) [20] and with satisfactory knowledge and understanding of the Greek language were included in the study. Those with an inadequate understanding of the Greek language and those who suffered from a psychiatric disorder were excluded from participating in the study.

In addition, 60 matched controls, namely, healthy adult and sexually active women, with adequate knowledge of writing and understanding of the Greek language were recruited. Healthy women were sought from the survivor’s social environment but not including their partner, as this would have influenced the responses. The researcher asked the survivor to suggest women from her environment contribute to the study as a healthy population.

### 2.3. Measurements

Survivors and controls completed demographic, clinical, and other data and the Female Sexual Function Index (FSFI). The FSFI is a 19-item, self-report questionnaire that addresses six domains of women’s sexual function; desire, arousal, lubrication, orgasm, satisfaction, and pain [21]. It has been validated and used in cancer survivors [22]. The questions refer to the sexual function of the last month and each question receives a single answer. Some items refer to frequency, and responses include “No sexual activity” (score 0) and a five-point scale, with responses ranging from “almost never or never” (score 1) to “almost always or always” (score 5). Other items assess intensity, and responses include “no sexual activity” (score of 0) and a five-point scale, with responses ranging from “very dissatisfied” (score of 1) to “very satisfied” (score of 5). The items that refer to pain or discomfort during or after vaginal penetration are reverse coded and higher scores indicate less pain or discomfort. The total score is calculated by adding the scores of the six subscales and is valid only for women who are sexually active in the last month. Higher scores indicate a greater degree of sexual function. The higher the score, the healthier the woman’s sexual function. Women with a score below 26.5 are likely to have some degree of sexual dysfunction and require clinical and laboratory investigation [21]. It is translated and validated in the Greek language [23]. The subscales of the FSFI had excellent internal consistency, based on the Cronbach alpha (desire = 0.964, arousal = 0.974, lubrication = 0.984, orgasm = 0.973, satisfaction = 0.977, pain = 0.975, total score = 0.987).

### 2.4. Ethics

The research was performed after we were granted permission from the hospitals’ and university’s ethics and research committee. Participants were informed verbally and in writing about the aim of the study, anonymity, confidentiality, voluntary participation, and the possibility of withdrawing from the study at any time before they signed the consent form. The participants subsequently completed the questionnaires in the presence of the researcher, after a thorough explanation. In addition, the protection of the participant’s personal data was ensured by the anonymous completion of questionnaires and code assignment. Permission was also granted to derive additional clinical data from the survivors’ medical records for the purposes of the study.

### 2.5. Statistical Analysis

Data were expressed as mean ± SD (standard deviation) for the quantitative variables and as frequencies and percentages for the qualitative variables. The Kolmogorov–Smirnov test was used for the normality analysis of the quantitative variables.

The comparisons of quantitative and categorical demographic variables between groups to test group homogeneity were performed using the Mann–Whitney and the Chi-square tests, respectively. Comparisons of the subscales of the FSFI between the survivors and controls were performed using the Mann–Whitney test, because these data did not follow a normal distribution.

Unifactorial analysis was performed using Spearman’s correlation coefficient, Mann–Whitney, and Kruskall–Wallis tests, to analyze the relationship between the dependent variable (total FSFI score) and the quantitative and qualitative demographic and clinical characteristics. All variables that yielded a *p*-value < 0.10, in unifactorial analysis, were then included in a multiple linear regression model using enter method to determine the independent predictors for women’s sexual function. All assumptions of linear regression analysis were examined (homoscedasticity, linearity, normality, and independence of error terms, as well as multicollinearity of independent variables).

All statistical analyses were performed using the statistical package SPSS version 21.00 (IBM Corporation, Somers, NY, USA). All tests were two-sided. A *p*-value < 0.05 was defined as the level of statistically significant difference. 

## 3. Results

### 3.1. Demographic and Clinical Data of Survivors

The sample consisted of 56 Greek women survivors with an average age of 44 years (age range 19–67 years). The mean age at transplantation was 41 years (range 19–63 years) and the mean time since transplantation was 3 years (range 1–5 years). Most survivors were married (73.2%), had children (66.1%), were university graduates (51.8%), were employed (64.3%), and 50% of them were postmenopausal. Functional status was ECOG 0 in 80.4% (Table 1).

Regarding their diagnosis, 48.2% of survivors had Hodgkin Lymphoma (HL), 32.1% Non-Hodgkin Lymphoma (NHL), and 19.6% had Multiple Myeloma (MM), with 41.1% having at least one disease relapse. All survivors had received chemotherapy, 32.1% radiotherapy, 33.9% immunotherapy, and 10.7% targeted therapies. Moreover, 14.3% of the women reported developing a musculoskeletal problem after the treatments and AHSCT (Table 1).

The groups of survivors and controls were homogeneous with respect to demographic and clinical characteristics (Table 1), except in the presence of musculoskeletal problems (*p* = 0.048) and employment, as most of the surviving women were retired.

### 3.2. Comparison of Sexual Function between Groups

As shown in Table 2, women survivors had an average level of sexual dysfunction [total score mean ± SD = 22.51 ± 8.95 (min–max: 1–36)], as an FSFI score less than 26.5 is considered as having some degree of sexual dysfunction. The best sexual function domain was “pain” [mean ± SD = 4.60 ± 1.54 (min–max: 0–6)], and the most affected was “orgasm” [mean ± SD = 3.19 ± 1.72 (min–max: 0–6)].

When the FSFI scores were compared between survivors and controls (Table 2), it was observed that controls had statistically significantly higher scores in the subscales, “desire” (*p* = 0.006), “arousal” (*p* = 0.001), “lubrication” (*p* = 0.002), “orgasm” (*p* = 0.001), “satisfaction” (*p* = 0.002), “pain” (*p* < 0.0005), as well as in the total FSFI score (*p* < 0.0005).

### 3.3. Unifactorial Analysis of Survivors’ Demographic and Clinical Characteristics in Relation to Total FSFI Score

The unifactorial analysis of the total FSFI score in relation to the survivors’ demographic and clinical characteristics is presented in Table 3. There was a statistically significant correlation between the total FSFI score and age (*p* < 0.0005).

Women survivors with a functional status ECOG 0 (*p* < 0.0005), who did not have children (*p* = 0.002) and who were premenopausal (*p* < 0.0005) had a higher total FSFI score, compared to those whose functional status was ECOG 1, who had children or were postmenopausal. Regarding the diagnosis, there was a statistically significant difference between women with MM and those with lymphoma (*p* < 0.05). In addition, those survivors who had attended high school had lower total FSFI scores compared with those who had attended university or had received post-graduate education (*p* = 0.002).

### 3.4. Multifactorial Analysis of Survivors’ Demographic and Clinical Characteristics in Relation to Total FSFI Score

Table 4 presents the multifactorial analysis, which was conducted using the total FSFI score as a dependent variable and age, functional status (ECOG), existence of children, education, diagnosis, and menopausal status (all variables with *p* < 0.1 in the unifactorial analysis), using the enter method. Since age, functional status (ECOG), and menopausal status showed collinearity with the other variables, these variables were excluded from the analysis. All remaining variables accounted for 55.0% of the variance in the total FSFI score [*R*^2^ = 0.550; *F* (7,51) =15.56, *p* < 0.005]. According to the results of the analysis, only survivors’ age showed a statistically significant effect on the total FSFI score, explaining 52% of the variance (Beta coefficient ± SE: −0.54 ± 0.10; *p* < 0.005), meaning that increasing age was associated with a reduction in sexual function.

## 4. Discussion

Women survivors after AHSCT had an average level of sexual dysfunction. Age was the strongest factor affecting survivors’ sexual function. The total FSFI score was also significantly associated with functional and menopausal status, the presence of children, education, and diagnosis.

Bersvendsen et al., (2021) assessed 110 women, survivors of lymphoma and AHSCT (median time from AHSCT, 9 years), using the Sexual Adjustment Questionnaire (SAQ) [24]. Similar to our findings, age significantly affected sexual function, with older women being less likely to be sexually active and premenopausal women being more sexually active. Their sample had a good level of sexual functioning, with the domain of sexual activity most negatively affected and the domain of pain and pleasure least affected. Tierney et al., (2015) studied 63 premenopausal women one year after HSCT [25]. Their mean age was 35 years and most of them had HL and had undergone AHSCT. FSFI scores showed that they had low desire and arousal, lubrication, absent or infrequent orgasm, painful intercourse, and overall dissatisfaction with their overall sex life (all at lower scores than our findings). This finding can be attributed to the fact that the researchers assessed women’s sexual function only one year after HSCT. Nevertheless, it is reported that some recovery of sexual function could occur during the first two years after HSCT, but survivors are still more likely than healthy people to report some kind of sexual dysfunction, up to 10 years later [16,17].

The relationship between increasing age and reduced sexual function has been studied and known for several years, both in the general population [26] and in previous studies with a mixed sample of male and female survivors of hematologic malignancies and autologous or allogeneic HSCT [18,27,28]. The normal aging process also negatively affects sexuality and often, older women can feel sexually undervalued. However, many older women remain sexually active and happy, provided they have a willing and able partner [29].

Contrary to the findings of this study, studies note that employment [18], radiation therapy [24,27], and time since transplantation [25] affected sexual function, while diagnosis did not [30]. Furthermore, in 35 women lymphoma survivors, with a mean age of 32 years, who had not undergone AHSCT, sexual dysfunction was reported in only 31% and was not significantly associated with age or treatment. The majority of these women had a total FSFI score greater than 26 [31]. So, AHSCT seems to be an additional aggravating factor in sexual function. Menopausal status was also not associated with sexual function in the Beckjord et al., (2011) study of NHL survivors [32], neither was age or years since diagnosis in the Recklitis et al., (2010) study of HL survivors [33].

Regarding women’s menopausal status, this was found to correlate with sexual function, with postmenopausal women being significantly worse. Natural menopause is a developmental process in women’s lives, that also affects sexuality, sometimes positively and sometimes negatively, with studies indicating decreases in sexual frequency, desire, and other changes, which may not always be perceived negatively by women, but rather as expected changes associated with aging [29]. In the general population of older women, decreased sexual interest and decreased arousal, vaginal dryness, dyspareunia and inability to orgasm, are more likely to occur due to lower testosterone levels and postmenopausal changes [34], age, and decreased estradiol levels [35]. In AHSCT, premature ovarian failure and induced menopause are common late effects related to the intensity of the preparative chemotherapy regimen, radiation, and estrogen-dependent changes in the hypothalamic-pituitary-gonadal axis [36,37]. Women who have received chemotherapy and immunosuppressive therapy for hematologic malignancies have impaired sexual function secondary to early menopausal symptoms [25,38].

Women with MM had significantly worse sexual function than those with NHL or HL. MM is a disease with many persistent symptoms and complications, including sexual difficulties, even after treatments and AHSCT, which hamper the quality of life of survivors [39,40]. The most commonly reported symptoms are pain, fatigue and peripheral neuropathy, leading to significantly reduced patient functionality [41]. MM survivors identified many barriers to sexual activity related to the nature of their disease and sexual function problems, such as low desire, orgasm problems, vaginal dryness, and dyspareunia [42]. Survivors of MM reported worse quality of life than survivors of lymphomas [40] and worse than other patients with hematologic malignancies treated with HSCT [43]. Therefore, MM survivors will also report worse sexual function, since this correlates with the quality of life after HSCT [11]. Moreover, the sexual function of women with MM may be negatively affected by other factors, not directly related to the disease, such as older age (the mean age of women with MM was 55 years old) and the additional morbidity that derives from it.

In addition, education had a statistically significant effect on the total FSFI score. In cancer patients, a low educational level is associated with high symptom burden and distress and this is attributed to less adaptability of these patients [44]. Healthy married women in Turkey with lower educational levels had higher sexual dysfunction than those with higher education. This could be explained by the fact that a higher educational level could mean that women are more aware of their physical, psychological, and sexual situation and they tend to ask for support [45].

Additionally, having children was negatively associated with sexual function. Childbirth often leads to changes in a woman’s sexuality and sexual relationships. A decrease in the frequency of sexual intercourse and a reduced desire exist after pregnancy and continue into the breastfeeding period [29]. After these periods, becoming a parent changes one’s outlook on life and is also described as a life crisis. For women, maternity is an emotional and physical challenge, since they tend to take primary responsibility for the child. In addition, the transition from companionship to parenthood can be associated with feelings of anxiety, increased fatigue, decreased self-esteem, and a deterioration in relationship quality that often occurs with decreased couple communication, decreased sexual activity, desire, and satisfaction [46]. All these, combined with intensive treatments and the subsequent rehabilitation after AHSCT, may negatively affect women’s sexual life.

Long-term survivors of HSCT report musculoskeletal problems like muscle weakness, cramps, myalgia, and arthralgia [47]. Our findings indicated that 14.3% of women developed a musculoskeletal problem after the treatments and AHSCT. While, in some cases, these problems existed prior to AHSCT and continued in the recovery period, others were probably induced by the treatment with alkylating agents, radiotherapy, or long-term immunosuppression [16,47]. Still, the assessment and management of the musculoskeletal problems of HSCT survivors need to be further studied. In women, musculoskeletal problems like decreased mobility, neck, back, and joint pain, and associated changes in sensations and comfort, may limit sexual activity and, therefore, negatively affect their sexual function [48].

In the general population, approximately 40–45% of adult women have at least one manifestation of sexual dysfunction [49]. Among HSCT survivors, 26–83% of women report at least one manifestation of sexual dysfunction [17,50]. Our findings demonstrated a worse sexual function of survivors than healthy women with similar findings to Bersvendsen et al., (2021) who compared 110 women survivors with 550 healthy women [24]. Other studies, which included a sample of both men and women with hematologic malignancies and autologous or allogeneic transplantation, found that they had impaired sexual function when compared with controls [16,18].

It should be noted that the measurement tool that was used in this study, the FSFI, had excellent internal consistency. The FSFI is the most widely used tool for the multidimensional assessment of female sexual function [51] in sexually active heterosexual women and cancer survivors [52]. Its use has become the “gold standard” for assessing female sexual dysfunction [51].

Limitations of this study include its cross-sectional design, the sampling technique, and the small sample. The strict inclusion criteria, the few centers of HSCT in Athens, and the COVID-19 pandemic, with the restrictions on social life and in hospitals, also contributed to limited participants. Yet, the response rate was high due to the careful approach of women, alongside their doctor or nurse. The measurement tool used to assess sexual function is valid and reliable and it is the first study in Greece that dealt with sexual function after AHSCT and one of the few that exist in contemporary world literature.

## 5. Conclusions

To conclude, women survivors of hematologic malignancy and AHSCT had an average level of sexual dysfunction and significantly worse sexual function than healthy women. The survival from hematologic malignancies and AHSCT is now increasing, and more emphasis needs to be placed on long-term complications, such as sexual dysfunction. Evidence shows that sexual dysfunction is one of the most prevalent and persistent long-term problem after AHSCT that affects quality of life, but there are still gaps in research that need to be filled. The current world literature is limited in examining the sexuality issues of survivors after AHSCT. More research is needed and can be oriented to include longitudinal or mixed methods design, more diverse samples, interventions to address specific sexual dysfunctions, and explorations of pathophysiological mechanisms. The present study is the first study conducted in Greece and aspires to be a starting point for future studies on sexual function and AHSCT. The sexuality of survivors with hematologic malignancies and AHSCT is an important parameter of their quality of life that needs further research and the raising of awareness among healthcare professionals.

## Figures and Tables

**Table 1:** Demographic and clinical data of survivors and controls.

		Controls		Survivors		*p*-Value
		*N*	%	*N*	%	
ECOG	0			45	80.4	-
	1			44	19.6	
Relationship Status ^2^	Married	48	80.0	41	73.2	0.102
	Committed relationship	9	15.0	15	26.8	
Existence of children ^2^	No	20	33.3	19	33.9	1.000
	Yes	40	66.7	37	66.1	
Cardiovascular problem ^2^	No	53	88.3	51	91.1	0.764
	Yes	7	11.7	5	8.9	
Respiratory problem^2^	No	57	95.0	55	98.2	0.619
	Yes	3	5.0	1	1.8	
Renal problem ^2^	No	60	100.0	55	98.2	0.483
	Yes	0	0.0	1	1.8	
Musculoskeletal problem ^2^	No	58	96.7	48	85.7	0.048
	Yes	2	3.3	8	14.3	
Thyroid problem ^2^	No	49	81.7	49	87.5	0.448
	Yes	11	18.3	7	12.5	
Menopausal status ^2^	Premenopausal	39	65.0	28	50.0	0.133
	Postmenopausal	21	35.0	28	50.0	
Educational level ^2^	High School	18	30.0	21	37.5	0.134
	University	27	45.0	29	51.8	
	Post-graduate	15	25.0	6	10.7	
Age ^1^	Mean ± SD	42.4 ± 10.7		43.9 ± 12.2		0.484
Age at AHSCT				40.6 ± 11.5		-
Years from AHSCT				3.1 ± 1.5		
Diagnosis	NHL			18	32.1	-
	HL			27	48.2	
	MM			11	19.6	
Disease relapse	No			33	58.9	
	Yes			23	41.1	
Chemotherapy	Yes			100	100.0	
Radiotherapy				18	32.1	
Immunotherapy				19	33.9	
Targeted therapies				6	10.7	

Note. SD: standard deviation, NHL: Non-Hodgkin Lymphoma, HL: Hodgkin Lymphoma, MM: Multiple Myeloma. ^1^ Mann–Whitney test. ^2^ Chi-square test.

**Table 2:** FSFI scores of survivors and controls.

FSFI	Controls	Survivors	*p*-Value
Desire ^1^	4.14 ± 1.42	3.42 ± 1.35	0.006
Arousal ^1^	4.43 ± 1.58	3.50 ± 1.63	0.001
Lubrication ^1^	4.69 ± 1.55	3.81 ± 1.78	0.002
Orgasm ^1^	4.21 ± 1.65	3.19 ± 1.72	0.001
Satisfaction ^1^	4.85 ± 1.50	3.99 ± 1.70	0.002
Pain ^1^	5.45 ± 1.19	4.60 ± 1.54	<0.0005
Total score ^1^	27.79 ± 8.14	22.51 ± 8.95	<0.0005

Note. All variables were presented as mean ± SD (standard deviation). ^1^ Mann–Whitney test.

**Table 3:** Unifactorial analysis of survivors’ total FSFI score with the demographic and clinical characteristics.

		Mean ± SD	*p*-Value
ECOG ^1^	0	25.36 ± 7.01	<0.0005
	1	10.84 ± 6.20	
Relationship status ^1^	Married	21.72 ± 7.99	0.114
	Committed relationship	24.66 ± 11.22	
Existence of children ^1^	No	27.03 ± 8.47	0.002
	Yes	20.19 ± 8.39	
Employment ^1^	Employed	23.57 ± 7.77	0.338
	Unemployed-Retired-Housework	20.60 ± 10.73	
Educational level ^2^	High School	16.96 ± 8.89	0.002
	University	25.65 ± 7.02	
	Post-graduate	26.77 ± 9.05	
Diagnosis ^2^	NHL	20.30 ± 10.53 *	0.003
	HL	26.67 ± 5.32 *	
	MM	15.91 ± 8.70	
Disease relapse ^1^	No	24.12 ± 8.31	0.100
	Yes	20.20 ± 9.52	
Radiation therapy ^1^	No	21.82 ± 10.04	0.854
	Yes	23.96 ± 6.08	
Cardiovascular problem ^1^	No	22.85 ± 9.18	0.164
	Yes	19.02 ± 5.74	
Menopausal status ^1^	Premenopausal	28.35 ± 4.56	<0.0005
	Postmenopausal	16.67 ± 8.48	
Age	Spearman correlation coefficient	*R* = −0.765	<0.0005
Years from AHSCT		*R* = −0.164	0.227

Note. SD: Standard deviation, NHL: Non-Hodgkin Lymphoma, HL: Hodgkin Lymphoma, MM: Multiple Myeloma.* *p* < 0.05 vs. MM, ^1^ Mann–Whitney test, ^2^ Kruskal–Wallis test.

**Table 4:** Multifactorial analysis of survivors’ total FSFI score with demographic and clinical characteristics.

	Reference	Βeta	Standard Error	Standardized Βeta	*p*-Value
Constant		40.57	6.61		<0.005
Age	---	−0.54	0.10	−0.73	<0.005
Children (Yes)	No	2.73	2.26	0.15	0.234
Education (University-Postgraduate)	High School	2.50	2.15	0.14	0.251
Diagnosis (MM)	NHL/HL	−0.22	2.41	−0.01	0.928

Note. NHL: Non-Hodgkin Lymphoma, HL: Hodgkin Lymphoma, MM: Multiple Myeloma. Multiple linear regression analysis using the enter method (all independent variables entered simultaneously).

## Data Availability

The datasets generated during and/or analyzed during the current study are available from the corresponding author upon reasonable request.
